# Social inequalities in self-rated health by age: Cross-sectional study of 22 457 middle-aged men and women

**DOI:** 10.1186/1471-2458-8-230

**Published:** 2008-07-08

**Authors:** Emily McFadden, Robert Luben, Sheila Bingham, Nicholas Wareham, Ann-Louise Kinmonth, Kay-Tee Khaw

**Affiliations:** 1 Institute of Public Health, University of Cambridge, UK; 2 MRC Dunn Nutrition Unit, Cambridge, UK

## Abstract

**Background:**

We investigate the association between occupational social class and self-rated health (SRH) at different ages in men and women.

**Methods:**

Cross sectional population study of 22 457 men and women aged 39–79 years living in the general community in Norfolk, United Kingdom, recruited using general practice age-sex registers in 1993–1997. The relationship between self-rated health and social class was examined using logistic regression, with a poor or moderate rating as the outcome.

**Results:**

The prevalence of poor or moderate (lower) self-rated health increased with increasing age in both men and women. There was a strong social class gradient: in manual classes, men and women under 50 years of age had a prevalence of lower self-rated health similar to that seen in men and women in non-manual social classes over 70 years old. Even after adjustment for age, educational status, and lifestyle factors (body mass index (BMI), smoking, physical activity and alcohol consumption) there was still strong evidence of a social gradient in self-rated health, with unskilled men and women approximately twice as likely to report lower self-rated health as professionals (OR_men _= 2.44 (95%CI 1.69, 3.50); OR_women _= 1.97 (95%CI 1.45, 2.68).

**Conclusion:**

There was a strong gradient of decreased SRH with age in both men and women. We found a strong cross-sectional association between SRH and social class, which was independent of education and major health related behaviors. The social class differential in SRH was similar with age. Prospective studies to confirm this association should explore social and emotional as well as physical pathways to inequalities in self reported health.

## Background

Self-rated health (SRH) refers to a single item health measure that asks individuals to rate their health as excellent, good, moderate or poor. SRH is generally considered to be a valuable source of data on subjective health status, and is popular due to its simplicity to collect. It declines with age and has strong associations with all-cause mortality that are not explained by existing disease. [1-3] Previous studies have reported a social class gradient in SRH but have not explored in detail the magnitude of this difference across classes nor the extent to which education or health related behaviours may explain such differences. We examined the association between SRH and occupational social class by age and gender, and the extent to which education and health related behaviours explain such relationships.

## Methods

### Sample

The study population is in Norfolk, United Kingdom and includes the city of Norwich as well as surrounding small towns and rural areas. The cohort was recruited from general practice age sex registers between 1993–1997 as part of the Norfolk component of the European Prospective Investigation of Cancer (EPIC-Norfolk).[4] Detailed descriptions of the study methodology have been reported previously.[5] Approval for the study was obtained from the Norfolk Local Research Ethics Committee. Altogether 77 630 participants were invited, and 30 445 gave informed signed consent and completed a detailed health and lifestyle questionnaire[5] Of these 25 639 men and women aged 39–79 years attended a health examination. Because we required participants who were willing to provide detailed information and participate in a long-term follow-up study, we only had a population response rate of about 45%, so participants were not a random population sample. Nevertheless, they were comparable to national samples with respect to many characteristics, but with a slightly lower prevalence of smokers.

### Measures

In the health and lifestyle questionnaire, participants were asked to assess their general health using the question "In general, would you say your health is?" with response options of "excellent, good, moderate or poor".[1]

Social class was classified according to the Registrar General's occupation based classification scheme across six categories.[6,7] Social class I consists of professionals, class II includes managerial and technical occupations, class III is subdivided into non-manual and manual skilled workers (III nm and III m), class IV consists of partly skilled workers, and class V comprises unskilled manual workers (the detailed classification is described elsewhere.[7]). For men, social class was coded using their own occupation except when they were unemployed in which case their partner's social class was used. Unemployed men without partners were unclassified. Last employment was used for men who were retired. Social class in women was based on their partner's except when the partner's social class was unclassified, missing, or they had no partner in which case social class was based on their own occupation. An unemployed woman without a partner was coded as unclassified.

Personal medical history was assessed using the question in the Health and Lifestyle Questionnaire, "Has the doctor ever told you that you have any of the following?" followed by a checklist of diseases including myocardial infarction, stroke, cancer and diabetes mellitus. Yes/no responses to the questions "Have you ever smoked as much as one cigarette a day for as long as a year?" and "Do you smoke cigarettes now?" were used to derive smoking history.[8,9] Alcohol consumption was derived from a food frequency questionnaire (FFQ) collected at the baseline clinic visit. The EPIC-FFQ comprised of a list of 130 foods. Under the "drinks" category, nine responses ranging from never to more than six times per day were given for four types of alcoholic drink: half pint of beer, lager or cider, a glass of wine, single unit of spirits (whisky, gin, brandy, vodka, etc.) and a glass of sherry, port, vermouth or liqueurs. Participants were asked to tick each category based on their average alcohol consumption in the previous year. Average alcohol consumption in units/week was calculated.[10,11] For the purpose of this study smoking status was re-categorised as current smokers and ex-/nonsmokers, and alcohol consumption was grouped as nondrinkers, people who drink 1–13.9 units/week, and people who consume ≥ 14 units/week.

Height and weight were measured by trained nurses with participants dressed in light clothing and with their shoes removed.[12] A stadiometer was used to measure height to the nearest 0.1 cm. Salter scales were used to measure weight to the nearest 100 g. Body mass index (BMI) was then calculated as weight (kg)/height^2 ^(m^2^). Body mass index was used as a proxy for poor diet.

Educational status was based on the highest qualification attained and was categorised into four groups: degree or equivalent, A-level or equivalent, O-level or equivalent, and less than O-level or no qualifications. O-level indicates educational attainment to the equivalent of completion of schooling to the age of 15 years and A-level indicates educational attainment to the equivalent of completion of schooling to the age of 17 years. This was regrouped into two groups: those who finished school (O levels or more), and those who did not (less than O level or no qualifications).

Habitual physical activity was assessed using two questions referring to activity during the past year. The first question asked about usual physical activity at work, classified as four categories: sedentary, standing (e.g. hairdresser, guard), physical work (e.g. plumber, nurse) and heavy manual work (e.g. construction worker). The second question asked about the amount of time spent in hours per week in winter and summer in other physical activity. A simple index allocated individuals to four ordered categories: inactive (sedentary job and no recreational activity); moderately inactive (sedentary job with < 0.5 hours recreational activity per day, or standing job with no recreational activity); moderately active (sedentary job with 0.5–1 hour recreational activity per day or standing job with < 0.5 hours recreational activity per day or physical job with no recreational activity); and active (sedentary job with > 1 hour recreational activity per day or standing job with > 1 hour recreational activity per day or physical job with at least some recreational activity or heavy manual job). This index was validated against heart rate monitoring with individual calibration in independent studies. [13-15] We have also previously reported that this four point index is inversely related to all cause mortality and cardiovascular disease incidence in the EPIC-Norfolk population in men and women across a wide age and social class range.[16]

### Statistical analysis

Descriptive statistics including means and percentages are used to show the characteristics of the study sample. Two sample *t*-tests were used to compare differences in mean values. The assumption of equal variances was verified. Differences in percentages were compared using χ^2^-tests. Analysis of variance was used to obtain mean values of each descriptive variable for each category of SRH. Differences in mean total values across the groups were evaluated using *F*-tests.

The proportion of men and women with a poor or moderate SRH was examined stratified by age group and social class. The relationship between self-rated health and social class was examined using logistic regression, with a poor or moderate rating as the outcome. We then examined odds ratios for poor or moderate health after adjusting for age and covariates BMI, smoking physical activity and alcohol consumption and educational status.

All statistical analyses will be performed separately for men and women using Stata version 8.0.

## Results

Of the 25 639 participants who attended the health check, 28 participants who were admitted to hospital for cardiovascular disease or cancer between agreeing to participate and attending the health check were excluded. A further 2422 participants who reported prevalent heart attack, stroke, and cancer at baseline; 261 with incomplete data on self-rated health; and 482 with no details of their last occupation were also excluded, leaving a total of 22 457 men and women in the current analyses.

Table 1 shows the sex-specific distribution of variables. Tables 2 and 3 show the descriptive characteristics of the cohort by social class in men and women respectively. Similar patterns were seen in both sexes for most variables. Mean age, BMI, and the proportion of current smokers all increased with decreasing social class from professional to unskilled. The proportion who finished school decreased with decreasing social class. The proportion of both men and women who did not drink alcohol at all increased with decreasing social class, while the proportion drinking over 14 units a week decreased with decreasing social class. The proportion of active and moderately active men increased with decreasing social class. More non-manual class men were moderately inactive compared to manual. The proportion of inactive women was greater in the manual classes while the proportion of moderately inactive was greater in the non-manual classes. A similar proportion of each class was classed as active.

**Table 1:** Descriptive characteristics of the EPIC-Norfolk cohort by sex measured at baseline from 1993–1997.

Variable		Variable distribution	
		
		Men	Women
N		10141	12316
Age (years)		58.4 ± 9.2	57.9 ± 9.2
Body Mass Index (kg/m^2^)		26.5 ± 3.3	26.2 ± 4.3
Cigarette smoking habit			
Never		34.5 (3477)	56.8 (6931)
Former		53.1 (5354)	31.9 (3891)
Current		12.4 (1249)	11.4 (1389)
Social Class			
I	Professional	7.8 (787)	6.5 (795)
II	Manager	38.2 (3875)	35.2 (4329)
III nm	Skilled non-manual	12.4 (1260)	19.6 (2419)
III m	Skilled manual	25.2 (2559)	21.4 (2634)
IV	Semi-skilled	13.4 (1362)	13.4 (1652)
V	Unskilled	2.9 (298)	4.0 (487)
Finished school		70.6 (7161)	53.6 (6599)
Self rate health			
Excellent		18.4 (1864)	16.1 (1983)
Good		63.9 (6478)	64.9 (7990)
Moderate		16.3 (1656)	17.6 (2166)
Poor		1.4 (143)	1.4 (177)
Units of alcohol per week			
0		9.5 (960)	16.8 (2074
1–13.9		63.4 (6432)	75.3 (9270)
≥ 14		27.1 (2749)	7.9 (972)
Exercise			
Inactive		29.3 (2966)	28.8 (3543)
Moderately inactive		24.7 (2505)	32.6 (4009)
Moderately active		23.3 (2367)	22.7 (2799)
Active		22.7 (2302)	16.0 (1965)

(Data are % (n) or mean ± s.d.)

**Table 2:** Descriptive characteristics of 10 141 men from the EPIC-Norfolk cohort by social class.

	Social class						P-value
		
	Professional	Manager	Skilled non-manual	Skilled manual	Semi- skilled	Unskilled	
N = 10 141	787	3875	1260	2559	1362	298	
Age (years)	57.8 ± 9.4	58.0 ± 9.3	59.5 ± 9.4	58.3 ± 9.2	59.1 ± 8.8	59.5 ± 8.7	< 0.001
Body mass index (kg/m^2^)	26.1 ± 3.2	26.5 ± 3.3	26.5 ± 3.3	26.5 ± 3.3	26.6 ± 3.4	26.6 ± 3.5	0.010
Cigarette smoking habit							
Never	49.4 (386)	38.2 (1473)	33.2 (415)	28.7 (729)	29.2 (394)	26.9 (80)	< 0.001
Former	45.3 (354)	51.6 (1991)	56.8 (699)	55.8 (1416)	54.6 (738)	52.5 (156)	
Current	5.3 (41)	10.2 (395)	11.0 (138)	15.6 (395)	16.2 (219)	20.5 (61)	
Finished school	96.9 (763)	82.0 (3176)	72.3 (911)	60.1 (1539)	49.1 (668)	34.9 (104)	< 0.001
Self-rated Health							
Excellent	27.1 (213)	22.4 (868)	17.7 (223)	13.0 (332)	13.8 (188)	13.4 (40)	< 0.001
Good	62.4 (491)	63.9 (2474)	66.0 (832)	64.7 (1656)	62.0 (845)	60.4 (180)	
Moderate	10.0 (79)	12.8 (497)	14.4 (182)	20.5 (524)	22.4 (305)	23.2 (69)	
Poor	0.5 (4)	0.9 (36)	1.8 (23)	1.8 (47)	1.8 (24)	3.0 (9)	
Units of alcohol per week							
0	7.5 (59)	6.9 (266)	8.9 (112)	10.8 (276)	14.7 (200)	15.8 (47)	< 0.001
1–13.9	60.1 (473)	60.3 (2338)	67.1 (845)	67.6 (1731)	63.1 (860)	62.1 (185)	
≥ 14	32.4 (255)	32.8 (1271)	24.1 (303)	21.6 (552)	22.2 (302)	22.2 (66)	
Physical activity							
Inactive	27.8 (219)	30.0 (1164)	36.9 (465)	25.9 (663)	27.4 (373)	27.5 (82)	< 0.001
Moderately inactive	37.7 (297)	29.8 (1156)	29.5 (372)	16.3 (416)	16.5 (225)	13.1 (39)	
Moderately active	20.3 (160)	22.0 (852)	18.3 (231)	26.3 (673)	26.7 (363)	29.5 (88)	
Active	14.1 (111)	18.1 (703)	15.2 (192)	31.5 (806)	29.4 (401)	29.9 (89)	

Data are % (n) or mean ± s.d.

**Table 3:** Descriptive characteristics of 12 316 women from the EPIC-Norfolk cohort by social class.

	Social class						P-value
		
	Professional	Manager	Skilled non-manual	Skilled manual	Semi- skilled	Unskilled	
N = 12 316	795	4329	2419	2634	1652	487	
Age (years)	56.6 ± 9.1	57.1 ± 9.3	59.7 ± 9.4	57.1 ± 8.9	58.4 ± 8.9	59.6 ± 9.1	< 0.001
Body mass index (kg/m^2^)	25.4 ± 4.3	25.8 ± 4.1	26.0 ± 4.2	26.5 ± 4.4	26.8 ± 4.6	27.5 ± 5.1	< 0.001
Cigarette smoking habit							
Never	63.4 (500)	57.4 (2462)	56.0 (1350)	55.9 (1459)	54.4 (887)	56.9 (273)	< 0.001
Former	29.0 (229)	32.6 (1397)	32.2 (776)	31.1 (813)	32.6 (532)	30.0 (144)	
Current	7.6 (60)	10.1 (431)	11.8 (283)	13.0 (340)	13.0 (212)	13.1 (63)	
Finished school	81.8 (650)	68.4 (2959)	52.5 (1271)	40.7 (1073)	32.3 (534)	23.0 (112)	< 0.001
Self-rated Health							
Excellent	21.9 (174)	19.5 (842)	15.8 (381)	13.6 (357	11.1 (183)	9.5 (46)	< 0.001
Good	66.2 (526)	65.0 (2817)	65.3 (1580)	65.4 (1722)	63.7 (1052)	60.2 (293)	
Moderate	108 (86)	14.3 (620)	17.8 (431)	19.4 (512)	23.2 (384)	27.3 (133)	
Poor	1.1 (9)	1.2 (50)	1.1 (27)	1.6 (43)	2.0 (33)	3.1 (15)	
Units of alcohol per week							
0	8.8 (70)	12.4 (537)	16.6 (401)	20.1 (529)	23.4 (387)	30.8 (150)	< 0.001
1–13.9	76.6 (609)	76.4 (3307)	76.8 (1858)	75.0 (1976)	72.3 (1194)	66.9 (326)	
≥ 14	14.6 (116)	11.2 (485)	6.6 (160)	4.9 (129)	4.3 (71)	2.3 (11)	
Physical activity							
Inactive	19.4 (154)	25.6 (1108)	34.6 (836)	28.8 (758)	31.3 (517)	34.9 (170)	< 0.001
Moderately inactive	35.5 (282)	34.0 (1471)	34.4 (831)	31.5 (829)	29.4 (485)	22.8 (111)	
Moderately active	28.8 (229)	24.3 (1050)	19.2 (464)	22.7 (598)	20.8 (343)	23.6 (115)	
Active	16.4 (130)	16.2 (700)	11.9 (288)	17.1 (449)	18.6 (307)	18.7 (91)	

Data are % (n) or mean ± s.d.

Table 4 shows that the prevalence of poor or moderate SRH increased with age in a similar manner in both men and women. The age gradient was similar in men and women with manual and non-manual occupations, however there was a large difference in prevalence of poor or moderate self-rated health between manual and non-manual classes. At all ages the prevalence of poor or moderate SRH was greater in the manual classes. The prevalence of poor or moderate SRH in men and women in non-manual social classes over 70 years was similar to that of men and women in manual social classes under 50 years. Table 4 also shows odds ratios of being in poor or moderate SRH in manual classes compared to non-manual classes for each age group. The odds of poor or moderate SRH are greater in manual classes at all ages, but the odds ratios remains similar for each age group.

**Table 4:** Prevalence and odds ratios of poor or moderate self-rated health by age group and social class in 10 141 men and 12 316 women from the EPIC-Norfolk cohort

Age group (years)	Proportion of poor or moderate self-rated health by social class, % (N)		Odds ratio#
Men N=10 141	Non-manual * N=5922	Manual * N=4219	

< 50	12 (157)	20 (170)	1.96 (1.55, 2.49)
50–54.9	14 (132)	21 (158)	1.72 (1.34, 2.22)
55–59.9	12 (107)	22 (154)	2.19 (1.67, 2.87)
60–64.9	15 (135)	27 (183)	2.00 (1.56, 2.57)
65–69.9	15 (136)	21 (136)	1.45 (1.12, 1.88)
≥ 70	17 (154)	29 (177)	1.97 (1.54, 2.52)

Total	14 (821)	23 (978)	1.87 (1.69, 2.08)

Women N=12 316	Non-manual * N=7543	Manual * N=4773	

< 50	13 (235)	20 (216)	1.74 (1.42, 2.13)
50–54.9	15 (197)	21 (191)	1.49 (1.19, 1.86)
55–59.9	17 (186)	25 (196)	1.64 (1.31, 2.06)
60–64.9	17 (186)	22 (157)	1.37 (1.08, 1.74)
65–69.9	18 (198)	25 (169)	1.55 (1.23, 1.95)
≥ 70	21 (221)	31 (191)	1.74 (1.39, 2.19)

Total	16 (1223)	24 (1120)	1.58 (1.45, 1.73)

* chi squared test between manual and non-manual classes, P < 0.001

# Odds ratio for poor or moderate self-rated health in manual classes compared to the baseline non-manual classes.

Table 5 shows the adjusted odds ratios of being in poor or moderate SRH by social class in men and women. There was an inverse association between odds of poor or moderate SRH and social class in both men and women, with similar odds ratios for men and women. Adjustment for behavioural factors BMI, smoking, physical activity and alcohol consumption attenuated the association in women, particularly in the lower social classes, whereas in men there was little effect on the association. Further adjustment for educational level attenuated the association in both men and women. However men and women in class V were still approximately twice as likely to report poor or moderate health than those in class I, after adjusting for age, BMI, smoking, physical activity, alcohol consumption and educational level. Models adjusted for each covariate individually are displayed in additional file 1: Table 6. In both men and women individual adjustment for smoking, alcohol intake and educational level each somewhat attenuated the association, while adjustment for BMI had little effect. Adjustment for physical activity strengthened the association.

**Table 5:** Adjusted odds ratios of being in poor or moderate self-rated health by social class in 10 141 men and 12 316 women from the EPIC-Norfolk cohort

		Odds ratio (95% CI)	
		
Social class		Men N=10 141	Women N=12 316
Model 1			
I	Professional	1.0	1.0
II	Manager	1.35 (1.06, 1.73)	1.34 (1.06, 1.69)
III nm	Skilled non-manual	1.61 (1.23, 2.12)	1.63 (1.28, 2.07)
III m	Skilled manual	2.43 (1.90, 3.10)	1.96 (1.55, 2.47)
IV	Semi-skilled	2.66 (2.05, 3.45)	2.42 (1.90, 3.08)
V	Unskilled	2.94 (2.09, 4.15)	3.07 (2.30, 4.09)

Model 2			
I	Professional	1.0	1.0
II	Manager	1.31 (1.02, 1.69)	1.24 (0.98, 1.57)
III nm	Skilled non-manual	1.47 (1.12, 1.94)	1.38 (1.08, 1.77)
III m	Skilled manual	2.49 (1.94, 3.21)	1.61 (1.27, 2.06)
IV	Semi-skilled	2.65 (2.03, 3.46)	1.92 (1.50, 2.47)
V	Unskilled	2.92 (2.05, 4.17)	2.26 (1.67, 3.05)

Model 3			
I	Professional	1.0	1.0
II	Manager	1.25 (0.98, 1.61)	1.20 (0.95, 1.52)
III nm	Skilled non-manual	1.37 (1.03, 1.81)	1.30 (1.01, 1.66)
III m	Skilled manual	2.22 (1.72, 2.87)	1.46 (1.14, 1.86)
IV	Semi-skilled	2.30 (1.75, 3.02)	1.71 (1.32, 2.21)
V	Unskilled	2.44 (1.69, 3.50)	1.97 (1.45, 2.68)

Model 1 – age adjusted

Model 2 – age, BMI, smoking, alcohol intake and physical activity adjusted

Model 3 – age, BMI, smoking, alcohol intake, physical activity and education adjusted

## Discussion

Our results provide further support for the association between SRH and social class. [17-19] As expected there was a strong gradient of decreasing SRH with age in both sexes. At all ages, the prevalence of poor or moderate SRH was greater in manual class men and women than the non-manual social classes. The prevalence of poor or moderate SRH in manual workers under 50 years of age is similar to that seen in non-manual workers over 70 years old. The social class SRH differential appears to remain similar with increasing age. After adjusting for the effects of age there was still evidence of a strong social class gradient in SRH in both sexes. Some of this association was explained by education and health related behaviours assessed through BMI, smoking, alcohol intake and physical activity, but after full adjustment for these covariates, unskilled workers were still more than twice as likely to report poor or moderate SRH compared to professionals (OR_men _= 2.44 (95%CI 1.69, 3.50); OR_women _= 1.97 (95%CI 1.45, 2.68).

Thus in this population of middle-aged men and women, there is a large social class differential in SRH. It is unlikely that variations in self-rated health can have led to the gradient in social class, although people with poor health do drift down the social class gradient. It is more likely that characteristics related to poor social circumstances affect people's perceptions of their health. This could represent a gradient in physical or emotional health, or could represent different social experiences related to perception of health in different social classes.

SRH is generally considered to be a valuable source of data on health status, popular due to its simplicity to collect and its strong association with future mortality.[1-3,20] The social class gradient for chronic diseases such as cardiovascular disease is well recognised. [21-27] Differences in SRH might therefore reflect difference in prevalent disease. Although in this study we excluded individuals who had known serious chronic diseases such as cardiovascular disease and cancer, it is possible that respondents were taking a range of other illnesses into account.

In any single index self-reported measure of health response styles and reference points against which health is judged may vary between respondents. [28-31] Individuals in different social classes with similar physical health status may thus have different reference levels and criteria against which they judge their health. However the direction of such variation would arguably be in the opposite direction to the associations shown, with people surrounded by others with illness likely to normalise rather than over report poor health, and those surrounded by affluence being more sensitive to nuances in fitness and behaviour. The odds ratios may thus be under estimates of the true association between social class and self-rated health.[32]

A number of qualitative studies have examined the processes through which individuals evaluate their health status.[31,33] It appears that there may be important differences in people's perception of health between socio-economic groups. Men and women from higher social groups appeared to use a larger number of factors when assessing their health, including aspects such as being fit and active and the absence of illness, as well as aspects of well-being such as happiness and feeling in control.[31] In this population the social gradient in SRH was still present after adjustment for health related behaviours BMI, smoking, alcohol intake and physical activity, all of which may be related to SRH.

Self-rated health appears to be strongly patterned by social occupational class. Prospective studies are needed to confirm that the association seen in cross sectional studies is causal and to assess the contribution of SRH to the mortality differentials seen across social classes. These studies should explore social and emotional, as well as disease pathways to social inequalities in self-rated health.

### Strengths and limitations

This study has a number of limitations. The cross-sectional design limits conclusions on causality. While we were able to examine how far educational level and health related behaviours might account for some of the socio-economic differential in SRH, we did not examine the roles of all potential factors, including a range of mental and physical illnesses, and social context, which might explain some of the difference. Individuals with major medical conditions that could potentially have confounded the relationship between SRH and social class were however excluded from the analyses.

Occupation details and self-rated health were both obtained at the baseline survey between 1993–1997. Some degree of inaccuracy in reporting or recording this information is inevitable, however it seems likely that misclassifications would be random, and random measurement error is likely only to attenuate any relationships, not produce spurious relationships. Some controversy exists over whether a woman's social class should be graded using her own occupation or that of her husband.[16,34-36] Arguments for grading according to a woman's own occupation are that the standard of living in the household may be influenced by a woman's earnings, or her job may expose her to health hazards.[37] However, no clear difference between the two measures has been shown in women aged > 60, and a stronger association was seen with husband's social class in women aged 20–59 years.[16,34,38] Thus husband's social class was considered an appropriate classification in this cohort.

Selection bias is unlikely to explain the observed association between social class and self-rated health within the population since it is unlikely that there was a differential response in that people in manual social classes with good health were less likely to participate or vice versa. The study population comprises of participants willing to complete detailed questionnaires and attend health checks. Nevertheless there was a wide range of social class in this cohort with a distribution similar to the national distribution.[6,39] and the cohort is similar to the general resident population in England in terms of anthropometric variables, serum lipids and blood pressure[5] and of physical and mental functional health,[40] although there were fewer current smokers. Excluding those with unclassified or missing data for SES or SRH could cause bias, but only if these people differed from those included in the study with respect to the relation between SES and SRH, which seems unlikely.

## Conclusion

In conclusion, our results show a marked social gradient in self-rated health, with the prevalence of poor or moderate health in men and women in manual social classes under 50 years of age similar to that seen in non-manual men and women over 70 years old. Even after adjustment for age, education and health related behaviours, there was still strong evidence of a social gradient, with men and women in unskilled occupations approximately twice as likely to report poor or moderate subjective health as those in professional occupation.

## Competing interests

The authors declare that they have no competing interests.

## Authors' contributions

KTK, SB, and NW are principal investigators in the EPIC-Norfolk population study. SB is responsible for the dietary measurements and analyses. RL is responsible for data management and computing and data linkages for post coding. ALK is principal investigator on self-rated health and well being NIHR National School of Primary Care Research. EM conducted the data analyses and wrote the paper with KTK with contributions from other co-authors.

## Pre-publication history

The pre-publication history for this paper can be accessed here:



## Supplementary Material

Additional file 1 Click here for file
