# Supplementary material for: Social inequalities in self-rated health by age: Cross-sectional study of 22 457 middle-aged men and women
Source: BMC Public Health. 2008 Jul 8;8:230. doi: 10.1186/1471-2458-8-230 (PMC2491612; doi:10.1186/1471-2458-8-230)
Supplement: Additional file 1 [file 1471-2458-8-230-S1.doc]

# Additional file 1

**Table 6 - Adjusted odds ratios of being in poor or moderate self rated health in 10 141 men and 12 316 women from EPIC-Norfolk 1993-1997**

| Social class | | Odds ratio (95% CI) | |
| --- | --- | --- | --- |
|  |  | Men  N=10 141 | Women  N=12 316 |
| **Age, BMI** | |  |  |
| I | Professional | 1.0 | 1.0 |
| II | Manager | 1.33 (1.04, 1.70) | 1.36 (1.07, 1.71) |
| IIInm | Skilled NM | 1.61 (1.22, 2.12) | 1.63 (1.28, 2.08) |
| IIIm | Skilled M | 2.40 (1.87, 3.07) | 1.91 (1.51, 2.43) |
| IV | Semi-skilled | 2.62 (2.02, 3.41) | 2.32 (1.81, 2.97) |
| V | Unskilled | 2.92 (2.06, 4.12) | 2.83 (2.11, 3.79) |
| **Age, smoking** | |  |  |
| I | Professional | 1.0 | 1.0 |
| II | Manager | 1.30 (1.02, 1.66) | 1.30 (1.03, 1.64) |
| IIInm | Skilled NM | 1.53 (1.17, 2.02) | 1.58 (1.24, 2.00) |
| IIIm | Skilled M | 2.27 (1.77, 2.90) | 1.89 (1.50, 2.40) |
| IV | Semi-skilled | 2.45 (1.89, 3.18) | 2.31 (1.81, 2.95) |
| V | Unskilled | 2.63 (1.86, 3.73) | 2.90 (2.17, 3.89) |
| **Age, alcohol intake** | |  |  |
| I | Professional | 1.0 | 1.0 |
| II | Manager | 1.36 (1.06, 1.74) | 1.29 (1.02, 1.63) |
| IIInm | Skilled NM | 1.57 (1.20, 2.06) | 1.52 (1.19, 1.93) |
| IIIm | Skilled M | 2.33 (1.82, 2.98) | 1.75 (1.38, 2.21) |
| IV | Semi-skilled | 2.53 (1.95, 3.28) | 2.13 (1.67, 2.71) |
| V | Unskilled | 2.79 (1.98, 3.95) | 2.58 (1.92, 3.45) |
| **Age, physical activity** | |  |  |
| I | Professional | 1.0 | 1.0 |
| II | Manager | 1.38 (1.08, 1.77) | 1.30 (1.03, 1.63) |
| IIInm | Skilled NM | 1.59 (1.21, 2.09) | 1.51 (1.19, 1.92) |
| IIIm | Skilled M | 2.85 (2.22, 3.65) | 1.88 (1.49, 2.38) |
| IV | Semi-skilled | 3.11 (2.39, 4.04) | 2.35 (1.84, 2.99) |
| V | Unskilled | 3.52 (2.48, 4.99) | 3.02 (2.25, 4.04) |
| **Age, education** | |  |  |
| I | Professional | 1.0 | 1.0 |
| II | Manager | 1.27 (0.99, 1.62) | 1.27 (1.01, 1.60) |
| IIInm | Skilled NM | 1.46 (1.11, 1.93) | 1.48 (1.17, 1.88) |
| IIIm | Skilled M | 2.10 (1.63, 2.69) | 1.69 (1.33, 2.14) |
| IV | Semi-skilled | 2.22 (1.70, 2.89) | 2.04 (1.59, 2.61) |
| V | Unskilled | 2.33 (1.64, 3.32) | 2.52 (1.88, 3.39) |
